# Seasonal variations in quantitative and qualitative sperm characteristics in fertile and subfertile stallions

**DOI:** 10.5194/aab-63-145-2020

**Published:** 2020-05-14

**Authors:** Yara Suliman, Frank Becker, Armin Tuchscherer, Klaus Wimmers

**Affiliations:** 1 Institute for Reproductive Biology, Leibniz Institute for Farm Animal Biology (FBN), Wilhelm-Stahl-Allee 2, 18196 Dummerstorf, Germany; 2 Institute of Genetics and Biometry, Leibniz Institute for Farm Animal Biology (FBN), Wilhelm-Stahl-Allee 2, 18196 Dummerstorf, Germany; 3 Institute for Genome Biology, Leibniz Institute for Farm Animal Biology (FBN), Wilhelm-Stahl-Allee 2, 18196 Dummerstorf, Germany; 4 Professorship of Animal Breeding and Genetics, Faculty of Agricultural and Environmental Sciences, University of Rostock, Rostock, Germany

## Abstract

Horses are seasonal breeders with a natural breeding season beginning in spring and extending through midsummer. In this study, quantitative and qualitative parameters of chilled stallion semen were compared between fertile and subfertile stallions and between the breeding and the non-breeding season. Semen quality parameters compared included ejaculate volume, sperm concentration, total sperm number, sperm morphology, and computer-assisted semen analysis (CASA)-derived sperm movement characteristics obtained from two groups of warmblood stallions (n=8; four fertile stallions and four subfertile stallions), which differ in the seasonal pregnancy rate 80 %–90 % (fertile) vs. 40 %–60 % (subfertile). A total of 64 ejaculates were collected from the stallions (n=8; four in the breeding season and four in the non-breeding season of each stallion). No significant differences in the semen quality parameters between the fertile and the subfertile stallions in the non-breeding season were observed. However, in the breeding season the proportion of morphologically normal sperm, total motility, progressive motility, average path velocity (VAP), and curvilinear velocity (VCL) were significantly higher in the fertile group (P<0.05) when compared with the subfertile group. In addition, a significant seasonal variation in the proportion of morphological normal sperm was found in the fertile group between the breeding and the non-breeding season (P<0.05). Moreover, significant seasonal variations (P<0.05) in CASA parameters of mean VAP, straight line velocity (VSL), and beat-cross frequency (BCF) were observed in the fertile and the subfertile stallions, which tended to be lower in the non-breeding season. In conclusion, differences between the fertile and the subfertile stallions were observed only in the breeding season, and a few of CASA-derived parameters seemed to be significantly lower during the non-breeding season in both the fertile and the subfertile stallions.

## 1 Introduction

In a variety of domestic animals, reproduction is influenced by seasonal variations (and linked changes in weather conditions) rather than by climate change. The seasonal changes affecting reproductive capacity are translated into remarkable increases in the size and the function of the reproductive organs in both male and female animals (Gerlach and Aurich, 2000). The environmental signals such as temperature and photoperiod interact with the endogenous mechanisms in which the reproductive activity of the seasonal breeders is affected (Aurich, 2011). Likewise, horses show increased reproductive activity during spring and summer when the temperature and the onset and length of daylight are very appropriate and the food is available, whereas these factors decreased reproductive activity in the winter months (Gerlach and Aurich, 2000; Pickett et al., 1976).

In stallions, seasonal variations were found to involve testosterone and luteinizing hormone (LH) concentrations, sexual behaviour, semen volume, sperm output, and sperm motility, which were found to be higher in spring and summer compared to winter. Mares also exhibit increased reproductive capacity in spring and summer, which is reflected in sexual behaviour, hormonal levels in the blood such as LH, follicle-stimulating hormone (FSH), and gonadotropin-releasing hormone (GnRH), and the size of follicles and ovary (Aurich, 2011; Altinsaat et al., 2009; Janett et al., 2003; Gerlach and Aurich, 2000; Clay and Clay 1992).

The aim of the present study was to characterize the seasonal variations in parameters of ejaculate quality and morphological and functional characteristics of chilled semen. In order to facilitate detecting seasonal effects these parameters were analysed in samples derived from fertile and subfertile stallions. The analyses covered ejaculate volume, sperm concentration, total sperm number, sperm morphology, and CASA-derived sperm movement characteristics.

## 2 Material and methods

### 2.1 Animals

All animal procedures were performed in relation to the general terms and conditions of the EU-stallion station of different German State studs.

For this study eight warmblood stallions of the breeds Hanoverian (n=4), Mecklenburg (n=2), and Oldenburg Warmblood (n=2) from two insemination stations in Germany were used. All stallions exhibited normal sexual behaviour, and the semen samples were collected during the routine breeding procedure. Accordingly, no issues regarding animal experimentation apply. The selected stallions were aged between 7 and 12 years (three were 12 years old, two were 7 years old, and three were 9, 10, and 11 years old, respectively) covered at least 10 mares in the season. All stallions were realigned in a regular semen collection regime during the breeding period. Stallions were housed indoors in boxes with straw under the influence of natural climate conditions and the availability of food and water.

### 2.2 Experimental design

A two-factorial design was used considering the effect of season and fertility. Therefore, stallions were divided into two groups, which differ in the seasonal pregnancy rate (the total number of mares getting pregnant divided by the total number of mares bred during the year), a fertile group (four stallions, 80 %–90 % pregnancy rate) vs. a subfertile group (four stallions, 40 %–60 % pregnancy rate). Ejaculates were collected weekly from the fertile and the subfertile stallions during the breeding and the non-breeding seasons. A total of eight ejaculates from each stallion was used (four in the breeding the season and four in non-breeding season). The collection period lasted one breeding season and one non-breeding season.

### 2.3 Semen collection, evaluation, and processing

The artificial vagina (Hannover model, Minitüb, Germany) was used to collect semen samples from the stallions used in the present study. Before starting the semen collections for this study, all stallions were sampled two to three times in order to minimize the extra-gonadal sperm reserves, which were stored in the epididymis. After semen collections, the gel was removed from each ejaculate using a gel filter, the volume was determined using prewarmed graduated cylinder, and the concentration was measured using a photometer (Minitüb, Germany). Then, the total sperm number in the gel-free volume was calculated by multiplying the semen volume and the sperm concentration. After that, the semen was diluted using a prewarmed skim-milk extender (Minitüb, Germany) to a constant sperm concentration of $100\times 10^{6}$ sperm mL${}^{-1}$. The extended semen was then cooled down to 4 ${}^{\circ }$C, divided into 10 mL tubes, and loaded into transport container, which keeps a constant temperature of 4 ${}^{\circ }$C. Finally, the container was transported to the laboratory in Dummerstorf for 3 hours.

### 2.4 Examination of chilled semen

Stallion sperm motility was estimated using a computer-assisted semen analyser (AndroVision, Minitüb, Germany). Various measures of a total and a progressive motility ratio as well as sperm velocities including VCL, VSL, and VAP in micrometres per second (µm s${}^{-1}$) were taken and reported with CASA. In addition to sperm velocities, the amplitude of lateral head displacement (ALH) in micrometres, BCF in hertz, linearity, (VSL / VCL) ×100, straightness, (VSL / VAP) ×100, and wobble, (VAP / VCL) ×100, were measured and reported with CASA. For motility analysis, diluted semen was further diluted in the laboratory to obtain sperm concentration of 25–$50\times 10^{6}$ sperm mL${}^{-1}$ using the same extender, which was used in the collection station to dilute the semen samples before being transported to the laboratory. After that, diluted semen samples were stored at room temperature for 10–15 min. Then, 2.8 µL of fresh semen was loaded into a single prewarmed Leja chamber with a 10 µm depth, and the slide was placed on a stage at 37 ${}^{\circ }$C. This was subsequently analysed under a microscope (Nikon SMZ800, Japan) using ×40 objective. The settings of CASA used were as follows: frame rate of 60 Hz, minimum cell size of 7 pixels, number of microscopic fields to analyse of 15 fields, temperature of 37 ${}^{\circ }$C, and a minimum number of sperm cells to examine of 1000 spermatozoa per semen sample. Sperm cells with straightness (STR) ≥70 % and VAP ≥50 µm s${}^{-1}$ were considered as progressive motile sperm.

Sperm morphology was assessed using eosin-nigrosin stain (Nidacon, Sweden). The percentages of morphologically normal sperm and percentages of sperm with specific morphological defects were determined using a microscope (Nikon UFX-DX, Japan) with ×1000 magnification. Semen smears were prepared by mixing 20 µL semen with the same volume of the eosin-nigrosin stain. After that, the mixture was put on glass slide. Finally, the smear was air dried. A total of 100 sperm cells in each ejaculate was examined and classified.

Sperm abnormalities were classified by recording the number of specific morphologic defects considering the following categories: acrosome defect (knobbed, roughed and detached acrosome), sperm head defect (microcephalic, macrocephalic, pyriform, nuclear vacuoles, and tapered sperm head), detached sperm head, midpiece defect (distal midpiece reflex, segmental aplasia of the mitochondrial sheath, dag-like defect, pseudo-droplet defect, corkscrew midpiece defect, disrupted sheet and stump tail), proximal plasma droplet, distal plasma droplet, tail defect (simple coiled tail with or without retention of cytoplasmic material), and duplications (head or tail).

### 2.5 Statistical analysis

Statistical analyses were performed using SAS software, version 9.3 for Windows, copyright SAS Institute Inc., Cary, NC, USA. Descriptive statistics and tests for normality were calculated with the UNIVARIATE procedure of Base SAS software. The data of the semen quality parameters were evaluated by repeated-measurement ANOVA using the MIXED procedure of SAS/STAT software. The model included the fixed effects of fertility (fertile group, subfertile group), season (breeding season, non-breeding season), and the interaction of fertility and season. Repeated measures (season and ejaculate) of the same stallion were taken into account by the REPEATED statement of the MIXED procedure using an unstructured covariance type (UN) for season and a compound symmetry type (CS) for ejaculate to calculate the blocks of the block diagonal residual covariance matrix as UN@CS where @ is the direct product. Least-square means (LSMs) and their standard errors (SEs) were computed for each fixed effect in the model, and all pairwise differences between LSMs were tested by the Tukey–Kramer procedure. The SLICE statement of the MIXED procedure was used for performing partitioned analyses of the LSMs for the two-way interactions. Effects and differences were considered significant if P<0.05.

## 3 Results

### 3.1 Investigation of semen quality parameters associated with high fertility of stallion as measured by pregnancy rate

To investigate if stallions presenting different seasonal pregnancy rates also differ in their semen quality profile, spermatozoal qualitative and quantitative parameters were compared between the fertile and the subfertile stallions in the breeding and the non-breeding seasons.

As shown in Table 1 no significant differences were observed in the parameters including ejaculate volume as well as sperm concentration and total sperm number in chilled semen between the fertile and the subfertile stallions in both the breeding and the non-breeding seasons.

**Table 1:** Ejaculate volume, sperm concentration, and total sperm number of chilled semen collected from fertile and subfertile stallions during breeding and non-breeding season (least-square means ±SE).

	Breeding season		Non-breeding season	
Ejaculate	Fertile group	Subfertile group	Fertile group	Subfertile group
parameter	(n=4)	(n=4)	(n=4)	(n=4)
Ejaculate	45.6 ± 4.8	43.6 ± 4.8	30.5 ± 3.4	27.4 ± 3.4
volume (mL)				
Sperm concentration	275.3 ± 55.9	233.2 ± 55.9	373.3 ± 50.4	347.1 ± 50.4
(10${}^{6}$ mL${}^{-1}$)				
Total sperm	12.0 ± 1.9	9.5 ± 1.9	10.6 ± 1.4	9.5 ± 1.4
number (10${}^{9}$)				

Parameters for sperm morphologic characteristics of the fertile and the subfertile stallions in the breeding and the non-breeding seasons are summarized in Table 2. Between stallion groups, percentage of morphologically normal spermatozoa was significantly higher in the fertile group when compared to the subfertile group (67.1±4.9 % vs. 37.7±4.9 %, P=0.016). This significant difference was observed only in the breeding season and only in the proportion of morphologically normal spermatozoa. No significant differences in the morphologic characteristics were found between the fertile and the subfertile stallions in the non-breeding season.

**Table 2:** Percentage of morphological intact and defect spermatozoa of chilled semen collected from fertile and subfertile stallions during breeding and non-breeding season (least-square means ±SE).

	Breeding season		Non-breeding season	
Morphology	Fertile group	Subfertile group	Fertile group	Subfertile group
parameter (%)	(n=4)	(n=4)	(n=4)	(n=4)
Normal sperm	67.1 ± 4.9${}^{a,c}$	37.7 ± 4.9${}^{b}$	47.3 ± 6.0${}^{d}$	30.2 ± 6.0
Acrosome defect	3.5 ± 1.4	6.4 ± 1.4	3.9 ± 1.1	4.6 ± 1.1
Head defect	4.1 ± 1.4	5.5 ± 1.4	3.6 ± 1.6	6.3 ± 1.6
Detached sperm head	1.6 ± 0.9	3.8 ± 0.9	2.5 ± 1.9	4.4 ± 1.9
Midpiece defect	16.8 ± 5.2	33.5 ± 5.2	31.1 ± 5.7	44.3 ± 5.7
Proximal plasma droplet	3.3 ± 1.8	4.8 ± 1.8	5.4 ± 2.7	3.7 ± 2.7
Distal plasma droplet	1.3 ± 1.7	4.0 ± 1.7	1.3 ± 1.2	3.1 ± 1.2
Tail defect	1.9 ± 0.7	3.7 ± 0.7	4.4 ± 1.2	2.9 ± 1.2
Duplications	0.4 ± 0.2	0.6 ± 0.2	0.4 ± 0.2	0.4 ± 0.2

Means noted with different letters are significantly different (P<0.05). ${}^{a,b}$ between fertile and subfertile groups. ${}^{c,d}$ between breeding and non-breeding seasons.

In the breeding season, there was a significant difference in the total and the progressive motility between the fertile and the subfertile stallions (81.0±3.6 vs. 61.5±3.6; P=0.028 and 68.2±3.7 vs. 46.5±3.7; P=0.025). In addition, there was a difference in the VAP and VCL, which were significantly higher in the fertile group in comparison to the subfertile group (77.4±2.3 vs. 65.8±2.3; P=0.027 and 129.2±4.1 vs. 111.2±4.1; P=0.046). Furthermore, no significant differences in VSL, STR, linearity (LIN), wobble (WOB), ALH, and BCF were observed between the fertile and the subfertile stallions in the breeding season.

In the non-breeding season, no significant differences were found between the fertile and the subfertile stallions in all CASA-derived sperm movement characteristics as shown in Table 3.

**Table 3:** CASA-derived sperm motility parameters of chilled semen collected from fertile and subfertile stallions during breeding and non-breeding season (least-square means ±SE).

	Breeding season		Non-breeding season	
Motility	Fertile group	Subfertile group	Fertile group	Subfertile group
parameter	(n=4)	(n=4)	(n=4)(n=4)	
Total motility (%)	80.9 ± 3.6${}^{a}$	61.5 ± 3.6${}^{b}$	64.4 ± 4.7	45.8 ± 4.7
Progressive motility (%)	68.2 ± 3.7${}^{a}$	46.5 ± 3.7${}^{b}$	48.2 ± 4.4	27.9 ± 4.4
VAP (µm s${}^{-1}$)	77.4 ± 2.3${}^{a,c}$	65.8 ± 2.3${}^{b,c}$	57.4 ± 3.8${}^{d}$	49.2 ± 3.8${}^{d}$
VCL (µm s${}^{-1}$)	129.2 ± 4.1${}^{a}$	111.2 ± 4.1${}^{b}$	99.5 ± 7.3	86.4 ± 7.3
VSL (µm s${}^{-1}$)	55.9 ± 2.7${}^{c}$	46.8 ± 2.7${}^{c}$	39.9 ± 3.4${}^{d}$	32.1 ± 3.4${}^{d}$
STR (%)	71.4 ± 1.8	70.4 ± 1.8	68.1 ± 2.2	64.6 ± 2.2
LIN (%)	42.4 ± 1.4	41.4 ± 1.4	39.1 ± 1.2	36.6 ± 1.2
WOB (%)	59.4 ± 0.8	58.6 ± 0.8	57.3 ± 1.2	56.7 ± 1.2
ALH (µm)	3.7 ± 0.1	3.6 ± 0.1	3.4 ± 0.2	3.3 ± 0.2
BCF (HZ)	32.3 ± 0.5${}^{c}$	32.3 ± 0.5${}^{c}$	29.9 ± 0.7${}^{d}$	28.6 ± 0.7${}^{d}$

Means noted with different letters are significantly different (P<0.05). ${}^{a,b}$ between fertile and subfertile groups. ${}^{c,d}$ between breeding and non-breeding seasons.

### 3.2 Seasonal variations in quantitative and qualitative sperm characteristics of fertile and subfertile stallion groups

To investigate the seasonal variations in stallion semen characteristics, the differences in quantitative and qualitative parameters of stallion semen for each fertility group were examined. No significant seasonal variations were observed in ejaculate volume, sperm concentration, and total sperm number for the fertile and the subfertile stallion groups as shown in Table 1. The assessment of seasonal variations in stallion sperm morphology showed that the stallions in the fertile group have a great morphological similarity between the breeding and the non-breeding season, where the proportions of defect spermatozoa in chilled semen including acrosome defect, head defect, detached sperm head, midpiece defect, proximal and distal plasma droplets, tail defect, and duplications showed no significant differences between the breeding and the non-breeding season. However, the total proportion of morphologically intact spermatozoa in chilled semen was significantly higher in the breeding season (67.1±4.9 vs. 47.3±6.0; P=0.046). No significant differences between the breeding and the non-breeding season were observed in the proportion of morphologically normal spermatozoa or in all sperm defects in the subfertile group as presented in Table 2.

Furthermore, the percentage of the total and the progressive motility showed no significant differences between breeding and non-breeding seasons in both the fertile and the subfertile stallions. However, detailed analysis by a CASA system demonstrated a significantly higher percentage of VAP, VSL, and BCF in the breeding season when compared to the non-breeding season in the fertile and the subfertile stallions (P=0.014, 0.034, 0.030 in fertile group and P=0.038, 0.041, 0.001 in subfertile group). Moreover, no significant differences were found between the breeding and the non-breeding season for VCL, STR, LIN, WOB, and ALH in both the fertile and the subfertile stallions as summarized in Table 3.

## 4 Discussion

In the present study, quantitative and qualitative sperm characteristics of the fertile and the subfertile stallions were compared in the breeding and the non-breeding season to investigate which semen quality parameters are associated with high fertility of stallion as measured by pregnancy rate.

Ejaculate and morphological parameters were considered along with CASA-derived parameters including a series of variables which cannot be determined by the human eye (Gil et al., 2009; Quintero-Moreno et al., 2003). Accuracy and reliability of CASA measurement depend on the number of fields analysed, CASA settings, which differ from species to species, the chamber used, the temperature of measurement, and sperm concentration (Verstegen et al., 2002). Recently, Whitesell et al. (2019) compared the outcome of the traditional breeding soundness examination of stallions between the traditional and modern semen evaluation methods, suggesting that the use of modern techniques, such as CASA for evaluating sperm motility and differential interference contrast microscopy to evaluate sperm morphology, may give more accurate results about the fertility state of the stallion. They found that the use of modern evaluation techniques yielded in better estimates of sperm motility and morphology.

Regarding semen volume, sperm concentration, and total sperm number, the results of this study showed no significant differences between the fertile and the subfertile stallions in the breeding and the non-breeding seasons.

The proportion of morphologically normal spermatozoa found to be was significantly different between the fertile and the subfertile stallions in the breeding season. However, no significant differences were found between the fertile and the subfertile groups for acrosome defect, head defect, detached sperm head, midpiece defect, proximal plasma droplet, distal plasma droplet, tail defect, and duplications. In a previous study, in which sperm morphology was also associated with male fertility, a significant increase of morphologically normal spermatozoa was observed in the fertile stallions when compared to either the subfertile or the infertile stallions (Pesch et al., 2006; Neild et al., 2000). Whitesell et al. (2019) found also that the proportion of morphologically normal sperm as determined using differential interference contrast microscopy was the only single parameter found to be significantly correlated to stallion fertility. Jasko et al. (1990) reported a positive correlation between stallion fertility and normal sperm morphology, whereas Dowsett and Pattie (1982) and Voss et al. (1981) reported that there is no correlation between sperm morphology and stallion fertility. Moreover, Kavak et al. (2004) reported that the presence of some morphological sperm abnormalities belongs to normal stallion fertility. In several previous studies in which correlations between male fertility and sperm morphology were studied, conflicting results were observed. The results of the present study complement these reports in terms of discriminating seasonal effects. In fact, association between sperm morphology and fertility was detected for the proportion of normal sperm in the breeding season only, while in the non-breeding season no significant differences were observed in all morphology parameters between the fertile and the subfertile stallions.

Regarding sperm motility analysis, a significant difference was found in this study between the fertile and the subfertile group in the breeding season for some of the sperm movement characteristics derived from CASA including total motility, progressive motility, VAP, and VCL. Progressive sperm motility is one of the most important parameters used to determine stallion fertility (Jasko et al., 1992; Hurtgen, 1992). In fact, the percentage of progressively motile spermatozoa was found to be significantly higher in the fertile stallion in comparison to subfertile stallions (Neild et al., 2000). Using frozen stallion spermatozoa, Krik et al. (2005) found that the progressive motility and straightness of the spermatozoa were significantly correlated to first cycle fertility. However, Pesch et al. (2006) reported no significant correlation between total and progressive motility and fertility in stallion.

In this study, pattern of motility and velocities of sperm movement differed between the fertile and the subfertile stallions only in the breeding season. Detailed analysis by a CASA system demonstrated a significantly higher percentage of VAP and VCL in the fertile group when compared to the subfertile group. In the study of Broekhuijse et al. (2011), the CASA system was used to investigate the relationship between CASA parameters and boar fertility. Their results demonstrated that VAP was the only CASA parameter that showed a positive relationship with boar fertility. However, VSL showed a negative relationship to total number of piglets born. In contrast, Liu et al. (1991) found a positive correlation between VSL and human fertility, and the increases in VSL enable sperm to better fertilize the oocyte. Furthermore, Holt et al. (1985) found that the swimming speed (VCL) of ejaculated human spermatozoa was strongly correlated with in vitro fertilization rates. These conflicting data obtained from several studies could be related to different factors such as stallion, season, food quality, semen dilution, and CASA settings (Sieme et al., 2004; Davis and Katz 1992).

In the present study, the seasonal variations in quantitative and qualitative semen parameters for each stallion group were investigated. Seasonal variations in ejaculate volume, sperm concentration, and total sperm number, were not observed between the breeding and the non-breeding season for the fertile and the subfertile stallions. In contrast, Janett et al. (2003) reported that the gel-free volume was highest in spring and summer and lowest in winter. Regarding sperm concentration, they also reported that the highest sperm concentration obtained in autumn and the lowest in summer. In addition, Wach-Gygax et al. (2017) showed that the month of semen collection affected all semen quality parameters examined. They found that the volume of ejaculate was higher in summer compared to winter. In contrast, the sperm concentration was higher in winter compared to summer. However, in agreement to the results of the present study, Magistrini et al. (1987) suggested that there were no clear seasonal differences on stallion semen quality parameters.

Variations in seminal characteristics were also found to be influenced by ejaculate frequency and seasons. Increase in ejaculation frequency was associated with a decrease in sperm concentration and total sperm number (Pickett et al., 1975). The proportions of defective spermatozoa in chilled stallion semen including acrosome defect, head defect, detached sperm head, midpiece defect, proximal and distal plasma droplets, tail defect, and duplications showed no significant differences between the breeding and the non-breeding season.

The morphological examination of spermatozoa in chilled stallion semen revealed that the percentage of normal spermatozoa was significantly higher in the breeding season in the fertile stallion group (P<0.05). In the subfertile stallions a considerable morphological similarity between the breeding and the non-breeding season was obvious, with no significant differences in proportion of morphologically normal spermatozoa or percentage of defect spermatozoa observed. Results from routine semen quality analysis in the Netherlands showed more sperm abnormalities during the non-breeding season than in the breeding season (Van der Holst, 1975). In addition, Blottner et al. (2001) compared the quality and freezability of stallion semen during the breeding and the non-breeding seasons and found that higher values of morphologically intact spermatozoa were found in the non-breeding season. These findings are complemented by the results of the present study, where higher values of morphologically normal spermatozoa were observed in the breeding season but only in the fertile stallions.

Another important parameter for semen quality evaluation is sperm motility. For this purpose, a CASA system was used, which allows obtaining detailed and repeatable evaluations of sperm motility and velocity.

In the present study, significant seasonal variations in a part of CASA-derived sperm movement characteristics in the fertile and the subfertile stallions were observed. VAP, VSL, and BCF were found to be influenced by season and tended to be significantly lower in the non-breeding season in the fertile and the subfertile stallions. In addition, no significant differences were found in the total and the progressive motility or VCL, STR, LIN, WOB, and ALH between the breeding and the non-breeding season in the fertile and the subfertile stallions. Warnke et al. (2001) observed a higher percentage of motile spermatozoa during the non-breeding season compared to the breeding season. Furthermore, they found that the parameter BCF and mean sperm velocities were significantly reduced in the non-breeding season compared to the breeding season, which is confirmed by the findings of the present study.

There is clear evidence that the season affects sperm production and semen quality in stallions despite some conflicting results (Janett et al., 2003; Jasko et al., 1991; Magistrini et al., 1987, Pickett et al., 1975; Wach-Gygax et al., 2017). In the present study, seasonal variations in quantitative and qualitative semen parameters between the fertile and the subfertile stallions were investigated, where fertile stallions seem to be more influenced by seasons than subfertile stallions.

In the study of Roser and Hughes (1992), it was demonstrated that the basal secretion of testosterone, FSH, and LH in fertile stallion increased significantly in the breeding season. In contrast, the basal secretion of these hormones in the subfertile stallions did not significantly change between the breeding and the non-breeding season. This lack of seasonal differences in basal levels of testosterone, FSH, and LH in subfertile stallions may reflect the lack of seasonal regulation of the synthesis of gonadotropins (Roser and Hughes, 1992). This could explain the results of the present study, in which the subfertile stallions showed no significant differences between the breeding and the non-breeding season for most of the semen quality parameters studied. Furthermore, the significant increase in basal hormone concentrations in the fertile stallions between the breeding and the non-breeding season may trigger processes which lead to the increases in the proportion of morphologic intact spermatozoa in the breeding season compared to the non-breeding season.

## 5 Conclusions

This report complements previous studies by addressing the interaction of fertility and season while confirming that semen quality parameters are associated with fertility in stallions and that there are seasonal effects on semen quality parameters in stallions. In fact, two-factorial design of this study revealed that the effects of season on semen quality parameters, in particular on proportion of normal sperm, VAP, and VSL, were larger in fertile stallions. The results of the present study may give rise to important considerations in stallion management regarding the management of semen collection from stallions according to seasonal variations.

## Data Availability

The original data of the paper are available upon request from the corresponding author.

## Appendix A

**Table:**

ALH	Amplitude of lateral head displacement
ANOVA	Analysis of variance
BCF	Beat-cross frequency
CASA	Computer-assisted semen analysis
CS	Compound symmetry type
EU	European union
FSH	Follicle stimulating hormone
GnRH	Gonadotropin-releasing hormone
LH	Luteinizing hormone
LIN	Linearity
SAS	Statistical analysis system
SE	Standard errors
STR	Straightness
UN	Unstructured covariance type
VAP	Average path velocity
VCL	Curvilinear velocity
VSL	Straight line velocity
WOB	Wobble
